# Initiatives Addressing Precarious Employment and Its Effects on Workers’ Health and Well-Being: A Systematic Review

**DOI:** 10.3390/ijerph19042232

**Published:** 2022-02-16

**Authors:** Virginia Gunn, Bertina Kreshpaj, Nuria Matilla-Santander, Emilia F. Vignola, David H. Wegman, Christer Hogstedt, Emily Q. Ahonen, Theo Bodin, Cecilia Orellana, Sherry Baron, Carles Muntaner, Patricia O’Campo, Maria Albin, Carin Håkansta

**Affiliations:** 1 Unit of Occupational Medicine, Institute of Environmental Medicine, Karolinska Institutet, 11365 Stockholm, Sweden; bertina.kreshpaj@ki.se (B.K.); nuria.matilla.santander@ki.se (N.M.-S.); christer.hogstedt@gmail.com (C.H.); theo.bodin@ki.se (T.B.); cecilia.orellana@ki.se (C.O.); maria.albin@ki.se (M.A.); carin.hakansta@ki.se (C.H.); 2 MAP Centre for Urban Health Solutions, Li Ka Shing Knowledge Institute, Michael’s Hospital, Unity Health Toronto, Toronto, ON M5B 1W8, Canada; patricia.ocampo@unityhealth.to; 3 Lawrence S. Bloomberg Faculty of Nursing, University of Toronto, St. George Campus, Toronto, ON M5T 1P8, Canada; carles.muntaner@utoronto.ca; 4 Department of Community Health and Social Sciences, Graduate School of Public Health and Health Policy, City University of New York, New York, NY 10025, USA; emilia.vignola45@sphmail.cuny.edu; 5 University of Massachusetts Lowell, Lowell, MA 01854, USA; david_wegman@uml.edu; 6 La Isla Network, Washington, DC 20005, USA; 7 Division of Occupational and Environmental Health, Department of Family and Preventive Medicine, University of Utah School of Medicine, Salt Lake City, UT 84108, USA; emily.ahonen@utah.edu; 8 Center for Occupational and Environmental Medicine, Stockholm Region, 11365 Stockholm, Sweden; 9 Barry Commoner Center for Health and the Environment, Queens College, City University of New York, New York, NY 11367, USA; sherry.baron@qc.cuny.edu; 10 Dalla Lana School of Public Health, University of Toronto, Toronto, ON M5T 1P8, Canada; 11 Department of Mental Health, The Johns Hopkins University Bloomberg School of Public Health, Baltimore, MA 21205, USA; 12 Working Life Science, Karlstad Business School, Karlstad University, 65188 Karlstad, Sweden

**Keywords:** employment conditions, evaluation, health equity, implementation, informal employment, intervention, occupational health and safety, population health, precarious employment, worker health and well-being

## Abstract

The prevalence of precarious employment has increased in recent decades and aspects such as employment insecurity and income inadequacy have intensified during the COVID-19 pandemic. The purpose of this systematic review was to identify, appraise, and synthesise existing evidence pertaining to implemented initiatives addressing precarious employment that have evaluated and reported health and well-being outcomes. We used the PRISMA framework to guide this review and identified 11 relevant initiatives through searches in PubMed, Scopus, Web of Science, and three sources of grey literature. We found very few evaluated interventions addressing precarious employment and its impact on the health and well-being of workers globally. Ten out of 11 initiatives were not purposefully designed to address precarious employment in general, nor specific dimensions of it. Seven out of 11 initiatives evaluated outcomes related to the occupational health and safety of precariously employed workers and six out of 11 evaluated worker health and well-being outcomes. Most initiatives showed the potential to improve the health of workers, although the evaluation component was often described with less detail than the initiative itself. Given the heterogeneity of the 11 initiatives regarding study design, sample size, implementation, evaluation, economic and political contexts, and target population, we found insufficient evidence to compare outcomes across types of initiatives, generalize findings, or make specific recommendations for the adoption of initiatives.

## 1. Introduction

In recent decades, both precarious employment (PE) and the interest in this construct have increased significantly, especially in relation to large-scale economic and health crises and the consequences they have for workers in PE [1,2]. The PE construct continues to evolve, building on previous theoretical and empirical work [3,4,5,6,7,8,9]. While there is no generally accepted definition of PE, within the fields of public health and social or occupational epidemiology, this construct refers to several multifaceted elements concerning workers’ employment conditions. These characteristics include employment insecurity, inadequate levels of financial compensation or income volatility, and lack of rights and protections in the employment relation [10,11,12,13,14]. Employment insecurity is characterized by non-existent, temporary, seasonal, casual, or short-term contracts, underemployment, or the holding of multiple jobs [10,11,12,13,14]. The limited or missing worker rights and protections include a lack of unionization, social benefits and social protection, regulatory support, and an overall lack of workplace rights, which leaves precariously employed workers with low levels of control and a high risk of exploitation [10,12,14,15,16]. 

### 1.1. Links between PE, Non-Standard Employment (NSE), and Informal Work

In some instances, the constructs of PE and NSE are used interchangeably despite the many distinctions between them. The International Labour Organisation (ILO) defines NSE as employment that is not standard, which is typically understood as full-time, permanent, and based on a dependent relationship between an employee and an employer [12]. Aspects of NSE such as short-term or casual employment, part-time or on-call work, and self-employment are commonly linked to increases in employment and income insecurity [12]. Yet, they could also offer viable options and positive outcomes for workers in search of flexible work arrangements [12,17]. In many cases, however, NSE is not a preferred option for workers but a necessity due to a lack of standard forms of employment [12]. More, self-employment could conceal the existence of an employment relationship between those who pay for the work contracted and the alleged independent contractors, who in reality have only limited control over the conduct of the work [12]. While typically NSE is most often associated with insecurities, PE conditions could exist in both standard and NSE [12].

Similarly, PE conditions could exist in both formal and informal employment [12]. In low- or middle-income countries, the debates on employment arrangements and job quality are often framed around the informal or formal character of the work [12]. While there are differences between PE and informal work, there are also similarities. For instance, informal work is described as the inadequacy or lack of formal employment arrangements and is associated with instability given the high occurrence of temporary employment arrangements [12,18]. Such arrangements are also linked to underprivileged labour conditions, including limited legal protection of workers and lack of social and health benefits [12,18].

In this systematic review, we included initiatives addressing informal work in addition to those addressing PE. We made this decision given that there are a high number of workers in informal employment across the world (more than 60 percent in 2018 [19]) and concerns regarding employment security, income sufficiency, and rights and protections in the employment relation are shared by both precarious and informal workers [12,19].

### 1.2. Factors Contributing to the Rise in NSE and Implications for PE

What the ILO calls ‘the world of work’ has undergone significant adjustments in recent decades because of influences such as increased globalization, technological advances, social and climate change, and demographic fluctuations [12,20,21]. Not surprisingly, these occurrences gave rise to new opportunities but also new challenges, including the increased susceptibility of jobs to automation and digitalization, the transformation of existing occupations, and increases in NSE [20]. For instance, NSE forms such as temporary, part-time, temporary agency work, or self-employment are often adopted by companies in response to complex factors affecting their countries’ economies and labour markets [22]. While attempting to save costs and stay relevant in competitive business environments, companies may reorganize their business activities and hiring practices to enhance performance [20] and, as a result, switch from standard to NSE [22]. In other cases, companies use NSE to address short-term needs and/or seasonal spikes in production demands [12], accommodate workers who are voluntarily looking for more flexible work arrangements [20] or as an extensive probation period before moving workers into permanent employment [12,20]. In certain labour markets, the creation of part-time and temporary employment is regarded as a way to increase overall employment levels, increase the market participation of workers who have to balance work with unpaid family obligations, hold on to senior workers who may otherwise opt for retirement, or create opportunities for participation in the labour market for workers with limited levels of formal education [12,20]. 

Historically, NSE was more frequent in economic sectors known for the seasonal nature of their activities, such as construction, agriculture, transport, or the arts and entertainment industry, but in recent years, it has been adopted by other sectors such as the telecommunications or airline industry [12]. Individual companies’ decisions to use NSE is influenced by both intrinsic (e.g., size, industry, skill level required, etc.) and extrinsic factors [12].

When NSE is not a voluntary choice, it has the potential to increase workers’ insecurities regarding employment, earnings, and overall workplace rights [12] and, as a result, could increase PE. In addition, during certain phases of economic cycles, such as periods of crisis, these insecurities are accentuated, exposing workers in PE to even higher risks of unemployment, loss of income and, not surprisingly, to detrimental health effects [1,2]. Further, as pointed out by the ILO in its most recent global social protection report, the current pandemic is a stark reminder that the lack of social protections systems or the exclusion of workers in PE from such systems has implications that extend beyond individual worker health and business survival [23]. The PE conditions of many workers in NSE and especially their lack of employment and income security along with the lack of access to health and social protections could jeopardize public health and affect the overall functioning of our economies and societies [23]. 

### 1.3. Significance of PE to Public Health 

The literature examining the impact of various employment and work characteristics on workers’ health and well-being is extensive and diversified. Within this literature, a distinct body of research is preoccupied with explaining the effects of PE on workers’ physical and mental health [24,25,26,27,28], occupational health and injuries [29], and well-being [30,31], as well as on population-level health inequities [9,32,33,34,35,36,37]. Such studies show a clear link between PE and a range of negative health problems including mental [24,26,38,39,40], physical illnesses, well-being [31,41,42,43], occupation-specific afflictions [11,29], harmful lifestyle behaviours [8,21] and disparities in health care access [44].

The methodological approaches and indicators used to examine the impact of complex, cumulative, and life-time exposures to PE are continually expanded and refined [24,31,45,46,47,48,49]. An increasing number of studies acknowledge that certain population sub-groups have an increased exposure to NSE and PE. Several studies found that women, racialized groups, migrants, youth, and individuals with lower education are frequently found in both NSE and PE [12,50,51]. A study examining the effect of the 2008 crisis in Spain noted that the overall downward trend in self-employment was more pronounced for women [52]. Other research showed that (i) the prevalence of self-employment is higher for men [53,54], older workers and some immigrant groups [54], (ii) the reasons for opting for self-employment vary between genders [53,55], and (iii) the opportunities to succeed in entrepreneurial roles are impacted by factors such as gender and race [53]. As a result, more and more studies include disaggregated analyses to better understand the ways in which the differential exposure of worker sub-groups to forms of NSE and PE impact their health outcomes [6,8,36,51,56].

### 1.4. Review Justification, Contribution, Objectives, and Approach

The significant public health consequences of the multitude of PE-related problems speak to the importance and urgency of finding viable policy and practice solutions. This need is further confirmed by the increased prevalence of PE and intensification of aspects such as employment insecurity and income inadequacy triggered by the COVID-19 pandemic [1,23,57,58]. Having a clear understanding of the type of interventions that are effective and those that are not, and synthesising this information for policy makers, researchers, and workers’ and employers’ organizations, can support the planning of interventions to tackle PE. It is equally important to learn about the economic and political contexts in which interventions work, the ways in which different population sub-groups are affected by them, and the potential barriers and facilitators that could affect their implementation.

We conducted a preliminary search of PubMed to assess the availability of knowledge on interventions that could be used to counteract PE and its effects on worker health and well-being. We identified a knowledge gap and to address this gap, we undertook this systematic review. This review is the first to focus on initiatives specifically evaluated for effectiveness in addressing PE and its impact on the health and well-being of workers [59]. The findings of this review offer a snapshot of implemented and evaluated initiatives with potential to improve the health and well-being of workers in PE and inform several practice, policy, and research recommendations. In addition, the review summarizes several related gaps and offers solutions to address them. 

The specific objectives of our review were to identify, appraise, and synthesise existing research about “the effectiveness of initiatives aiming to or having the potential to eliminate, reduce, or mitigate workers’ exposure to PE conditions and its effects on the physical and mental health, safety, and well-being of workers and their families” [60]. The purpose of this manuscript is to synthesise the results pertaining specifically to studies that evaluated and reported health and well-being outcomes of initiatives. The results focused on other outcomes will be presented in separate manuscripts. This approach allows us to describe findings in more detail and share them with the research community earlier than would have been possible should we have waited for the complete analysis and synthesis of all studies, including those reporting outcomes that are not health specific.

We start by briefly describing the methods utilized to perform the systematic review. A more detailed description is available in the review protocol, published in the BMC Systematic Reviews journal [60] and its PROSPERO CRD42020187544 registration [61]. Next, we share the key findings with a focus on aspects such as the initiatives identified, their target economic sectors and population sub-groups, ways in which these initiatives could impact PE, the study design and data collection approaches used to assess the impact of initiatives, and the specific type(s) of PE and health-specific outcomes assessed and described. We discuss the findings through the perspective of their application, limitations, and identified gaps, along with an overview of some of the strengths and limitations of this review. We conclude with a discussion about practice, research, and policy implications. 

## 2. Materials and Methods

In keeping with our review protocol and to ensure consistency in approach and accuracy in reporting, the overall conduct of this systematic review and the reporting of findings is guided by the 2020 Preferred Reporting Items for Systematic Reviews and Meta-Analyses (PRISMA) framework [62]. This framework informed the development of the research questions, the systematic search process, the identification and selection of relevant studies, and the approaches used for quality appraisal, data extraction, and data analysis and synthesis.

While this manuscript is preoccupied solely with studies that evaluated and reported on health and well-being outcomes of initiatives addressing PE, this methods section summarizes the overall approach used to conduct the systematic review. 

### 2.1. Eligibility Criteria

The eligibility criteria were defined around the following considerations: population of interest, intervention(s) described, outcome(s) evaluated, study design, publication year, and language, as summarized next.

Population of interest: The population of interest consisted of workers (18 years of age and older, irrespective of gender, race, ethnicity, and migration status) and workers’ immediate or extended families. 

Initiatives examined: We defined initiatives as broadly as possible to include interventions, policies, legislations/regulations, programmes, guidelines, recommendations, collective agreements, or institutional practices. We included initiatives that were purposefully designed to address PE or that were designed for other purposes but that had the potential to address PE and/or its effects on the health and well-being of workers and their families. Initiatives had to be both implemented and evaluated and were considered regardless of the evaluation results (successful, unsuccessful, or inconclusive). 

Outcomes evaluated: The outcomes evaluated had to specifically consider changes to worker health and well-being whether or not they focused on changes in prevalence of PE and workers’ exposure to PE. 

Study design: We included qualitative, quantitative, or mixed-methods study designs and evaluations.

Publication year and language: We included studies published from January 2000 to May 2021, in English or another language spoken by members of our international review team: Catalan, Danish, Dutch, French, Italian, Norwegian, Romanian, Spanish, and Swedish. 

Exclusion criteria were:

(i) Editorial, commentary, discussion paper, review.
(ii) No clear initiative implemented.
(iii) Initiatives designed to:
  - Facilitate PE or increase exposure to PE.
  - Improve workers’ health through individual behavioural change without a focus on PE.
  - Improve work performance or health, safety, or well-being of workers with disabilities without a focus on PE.
  - Eliminate or reduce workers’ exposure to unemployment.
  - Eliminate, reduce, or mitigate the effects of unemployment on health and well-being.
  - Promote workers’ return to work after illness or injury without addressing PE.
(iv) Initiatives not evaluated formally or assessed using empirical data or initiatives with an evaluation that does not include a clear focus on reduction in precarious employment and/or on precarious workers and/or their families.
(v) Not in a language covered by the members of this team, mentioned for inclusion in the review protocol (Catalan, Danish, Dutch, French, Italian, Norwegian, Romanian, Spanish, and Swedish).

### 2.2. Search Strategies

**Figure 1:**
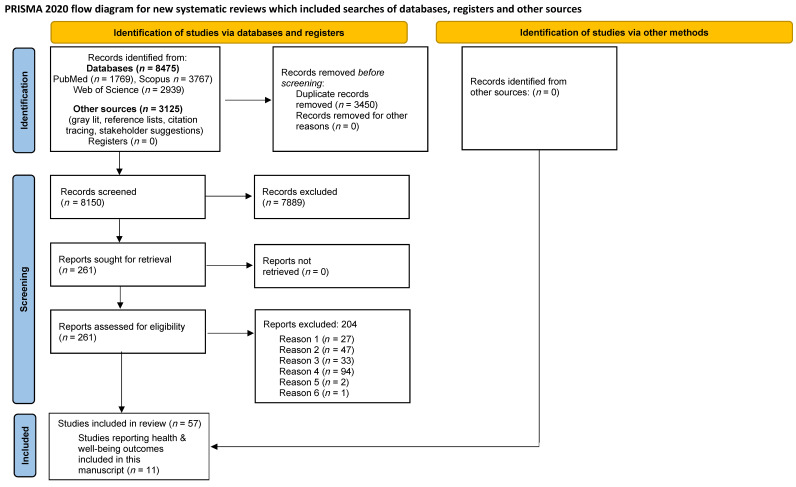
The flow diagram of study identification, screening, and inclusion. 1. Editorial, commentary, discussion paper, review; 2. No clear initiative implemented; 3. Initiative designed to (i) Facilitate PE or increase exposure to PE; (ii) Improve workers’ health through individual behavioural change without a focus on PE; (iii) Improve work performance or health, safety, or well-being of workers with disabilities without a focus on PE; (iv) Eliminate or reduce workers’ exposure to unemployment; (v) Eliminate, reduce, or mitigate the effects of unemployment on health and well-being; or (vi) Promote workers’ return to work after illness or injury without addressing PE; 4. (i) Not evaluated formally or assessed using empirical data or (ii) The evaluation does not include a clear focus on the reduction of PE and/or on precarious workers and/or their families. 5. Duplicate. 6. Not in a language mentioned in the protocol. PE—precarious employment.

Our search covered (i) three comprehensive and complementary academic databases: PubMed, Scopus, and Web of Science Core Collection and (ii) three sources of grey literature: the institutional databases of the International Labour Organization (ILO, Geneva, Switzerland), European Foundation for the Improvement of Living and Working Conditions (Eurofound, Luxembourg), and the Centers for Disease Control (CDC) and Prevention Community Guide of Evidence-Based Findings. Additionally, we reviewed the reference lists of included studies, conducted forward citation tracing, and consulted relevant stakeholders for suggestions. Specific details about the number of relevant studies identified, screened, and included are displayed in the PRISMA flow diagram (Figure 1).

### 2.3. Selection and Data Extraction 

Studies were selected for inclusion if they met all eligibility and none of the exclusion criteria listed in Section 2.1. The PRISMA flow diagram (Figure 1) outlines more details about the process of study selection. 

Twelve co-authors were involved as reviewers in the selection and data extraction process. After training sessions, pilot testing, and regular meetings to review progress and address unclarities, the first screening stage involved review of titles and abstracts against the eligibility criteria. Each title/abstract was screened independently by one reviewer. All studies suggested for inclusion underwent full-text review by two independent reviewers to confirm if they fit the inclusion criteria. Disagreements occurring during each stage were resolved through discussions among the two respective reviewers and/or discussions among other reviewers in the team. For studies confirmed for inclusion, data extraction was completed independently by the two reviewers, using a tailored data extraction form to capture all relevant information about each study.

### 2.4. Qualitative Assessment 

Given that eligible studies included a combination of qualitative, quantitative, and mixed-methods designs, we decided to use the Mixed Methods Appraisal Tool (MMAT) [63] to assess the methodological quality of included studies. The tool’s tailored assessment questions and selection algorithm facilitate classification of quantitative study designs and make it suitable for various study designs. The MMAT tool is not as detailed and extensive as other tools designed for qualitative and quantitative studies, such as the Critical Appraisal Skills Programme (CASP) Checklists [64], but its combination of tested usefulness, reliability, and ease of application with many heterogenous studies made it a robust choice [65,66]. For each eligible study, two reviewers used the tool independently and settled appraisal differences through discussions. To ensure that the inclusion and exclusion criteria were applied consistently by the large team of 12 reviewers involved in the full-text review stage, a core team of four reviewers involved in the quality appraisal process, performed another full-text screening of all studies initially deemed eligible before conducting the qualitative assessment. 

### 2.5. Presentation of Results

The results are presented using structured narratives and visuals (tables and maps), and organized around the study characteristics, target economic sector and population sub-groups, types of initiatives implemented, design and data collection approaches used to evaluate the impact of initiatives, ways in which the initiatives could impact PE, and health and well-being outcomes evaluated and reported.

## 3. Results

### 3.1. Search Results and Included Studies

Of the 11,600 potentially relevant studies identified, 8475 were found through the three academic database searches and 3125 through other sources, including review of the three institutional databases, review of reference lists, forward citation tracing, and consultation with stakeholders (Figure 1). After removing 3450 duplicate entries, we screened 8150 titles and abstracts and selected 261 studies for full-text review. Of these, 194 studies were determined to meet one or more of the exclusion criteria, listed in Figure 1, and were excluded at the full-text review stage, leaving 67 studies deemed eligible for inclusion. Data extraction was completed for all 67 studies. Following the added confirmatory step conducted before the qualitative assessment process, 10 more studies were excluded. As a result, 57 studies are included in the final systematic review. Of the 57 studies, 11 focus on health and well-being outcomes and are discussed in this manuscript. The remaining 46 will be summarized in a separate manuscript.

### 3.2. Study and Initiative Characteristics

A high-level overview of several key characteristics of the 11 studies is shown in Table 1. We review several of these characteristics in more detail next.

#### 3.2.1. Countries Examined, Type of Evidence, Study Design, Targeted Economic Sector, and Population Sub-Groups 

**Figure 2:**
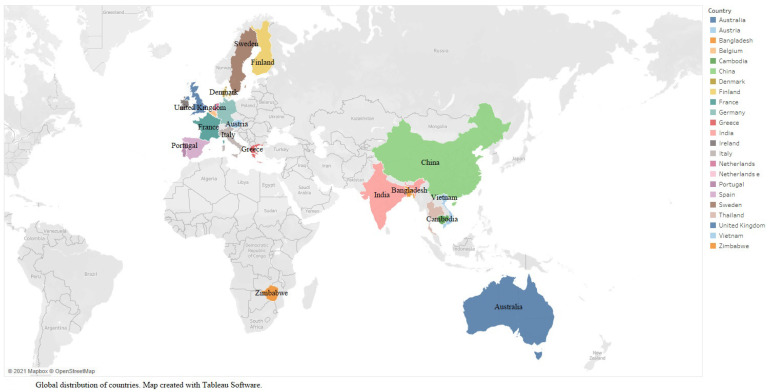
Global distribution of countries—22 countries across 11 studies.

The 11 studies reporting health and well-being outcomes, all published in English from 2000 to 2021, are listed in Table 2. A map of the global distribution of countries (1 country/study × 10 studies and 13 countries/study × 1 study) is displayed in Figure 2. The included countries represent four World Health Organization (WHO) regions—Africa [67], Europe [68,69,70], South-East Asia [71,72,73], and Western Pacific Region [74,75,76,77]—and a combination of low [67], lower-middle [72,73,74,77], upper-middle [71,76], and high-income countries [68,69,70,75]. Except for one institutional report [67] and one book chapter [74], all other studies were published as academic journal articles; these had a range of study designs, including five qualitative [67,70,71,75,77], one randomized controlled trial [69], one non-randomized [73], three quantitative descriptive [68,74,76], and one mixed methods [72]. This categorization of study designs uses the categories included in the Mixed Methods Appraisal Tool, 2018 version [63].

Eight initiatives were aimed at a particular sector, while three were not sector-specific [68,69,76]. Four initiatives addressed a single economic sector—agriculture [67], domestic services [70], sex industry [75], and textile industry [74]—while four studies addressed more than one sector, including retail, hospitality, agriculture, construction, and textile manufacturing [71,72,73,77]. 

The population sub-groups targeted in the studies were (i) informal workers—three studies [71,73,77], (ii) informal and formal workers employed in industries recognized for their high share of informal workers—three studies [67,70,72], (iii) workers in industries with a high share of PE—two studies [74,75], (iv) permanent and temporary workers—one study [68], (v) temporary and unemployed agency workers on sick leave—one study [69], and (vi) urban population sub-groups not covered by employment-based health insurance—one study [76].

#### 3.2.2. Description of Initiatives, Ways in Which They Could Impact PE, and Design and Data Collection Approaches Used to Evaluate Them

A range of initiative types are included in this review, reflecting not only the diversity and myriad components of PE and informal work that could lead to poor health but also the lack of standardized ways to tackle complex PE and informal work problems (Table 3). The initiatives encompassed the following: (i) adherence to international and national labour standards [74], (ii) employment protection legislation [68], (iii) international standards regulating product quality and production methods [67], (iv) a tax policy [70], (v) regulatory frameworks to govern an economic sector [75], (vi) two health insurance programs [73,76], (vii) a participatory process to involve workers in addressing and solving occupational health and safety risks [71], (viii) a participatory training program [77], (ix) a participatory return-to-work program [69], and (x) a program to recognize workers’ prior informal learning [72]. 

Of the 11 initiatives, only one [68] was designed specifically to address PE, through employment protection legislation for permanent workers and restrictions on the use of temporary employment. The other ten initiatives had the potential to address PE or its impact on the health and well-being of workers and their families. Based on our a priori classification of the ways in which an intervention could impact PE or its impact on the health and well-being of workers and their families, we found that the initiatives described in our included studies could work by: (i) limiting increases in the prevalence of PE [68]; (ii) eliminating, reducing, or mitigating workers’ exposure to PE and its impact on workers’ health and well-being [74,75]; (iii) eliminating, reducing, or mitigating workers’ exposure to informal employment or PE and their impact on workers’ health and well-being [67,70,72]; (iv) reducing and mitigating the effect of informal employment and PE on workers’ health and well-being [73,76], (v) mitigating the effect of informal employment and PE on workers’ health and well-being [71,77] and (vi) promoting workers’ return to work after illness or injury in a way that mitigates their PE [69].

Based on the type and scope of the 11 initiatives, only two had the potential to impact all three dimensions of PE identified in the 2020 Kreshpaj et al. systematic review of PE operationalizations [10], namely employment insecurity, income inadequacy, and lack of rights and protection in the employment relation [70,75]. One other initiative had the potential to impact two dimensions, employment insecurity and lack of rights and protection [68], while five initiatives had the potential to impact one dimension only: (i) employment insecurity [67], (ii) income inadequacy [72,74], or the lack of rights and protection in the employment relation [73,76].

The approaches utilized to evaluate the impact of initiatives differed greatly, often reflecting the specific study designs. First, there were considerable differences regarding sample size, with samples ranging from less than 35 individuals [67,75] to thousands [72,73] or hundreds of thousands of individuals [68,76], with important implications for the statistical power of the studies and the validity of their conclusions. Further variation came from the data collection methods, ranging from more objective and validated instruments [69,71] to use of secondary data [68,76]. There were also differences in approach employed to evaluate the outcome of initiatives. Several studies assessed outcomes by comparing differences among groups or sites that were exposed to an initiative versus those who were not [73,76], others compared the outcomes of different initiatives [68,75], and yet others compared outcomes before and after the adoption of an initiative [71,74]. In some cases, population sub-groups exposed to an implemented initiative along with experts on the topic were asked retroactively for their perception of the ways in which the initiative impacted certain outcomes [70,75,77]. Although one study utilized outcome indicators only [68], most studies collected and analysed both process and outcome indicators [67,69,70,71,72,73,74,75,76,77].

### 3.3. Health and Well-Being Outcomes 

While all 11 initiatives incorporated measures with the potential to improve the health and well-being of workers and their families, only eight also incorporated changes to employment conditions [67,68,70,72,73,74,75,76] (Table 4). As a result, these eight initiatives could have also reduced, eliminated, or mitigated workers’ exposure to PE or informal employment, in addition to addressing their health and well-being concerns. The three initiatives focused solely on changes to health and well-being outcomes [69,71,77] had the potential to mitigate the effect of PE or informal employment on workers’ health and well-being but given their lack of concern with changes in workers’ employment conditions, these lacked the potential to reduce, eliminate or mitigate workers’ exposure to PE or informal employment in the short-term.

To ensure that outcomes and the potential effectiveness of initiatives are interpreted in conjunction with the risk of bias assessments, the quality appraisal ratings are displayed in Table 4 along with the outcomes reported for each initiative. Further details about the quality appraisal results and the calculation of ratings are included in Section 3.5.

#### 3.3.1. Occupational Health and Safety 

Following the implementation and evaluation of initiatives, seven studies reported encouraging occupational health and safety outcomes [67,70,71,72,74,75,77], as detailed in Table 4. The most common occupational health and safety improvements reported were at the worker-level and consisted of (i) increased access to training, educational materials, and tools [70,77] and participation in training [70,77]; (ii) enhanced knowledge, attitudes, and behaviours about occupational health and safety issues [71,72]; and (iii) adoption of safer workplace practices [71,72]. A few studies indicated institutional-level changes, including (i) increased compliance with health and safety practices [75], (ii) improved access to welfare facilities [77], (iii) provision of health-related services to workers [67,74], and elimination of work hazards such as extreme temperatures and deficient lighting [71]. While most studies found positive impacts of the assessed initiatives, the study evaluating the impact of the Better Work program [74] did not show a clear relationship between the program exposure and specific outcomes, including exposure to extreme temperatures, concerns about dangerous equipment, and rates of accidents and injuries.

#### 3.3.2. Health and Well-Being 

Out of six studies describing health and well-being outcomes, four reported them for workers only [68,69,74,76] and two reported them for both workers and their families [67,73] (Table 4). Overall, except for one study that did not detect significant differences [69] and one study that reported both positive and negative outcomes [67], the reported worker health and well-being outcomes were positive [68,73,74,76]. With a few exceptions [67,68,69], these results were reflected in process indicators.

The outcomes measured were diverse, which is not surprising given the distinct initiatives assessed. For instance, one study evaluated the potential of a participatory program to address obstacles for return-to-work among unemployed workers and temporary agency workers, and examined participants’ functional status, pain intensity, perceived health, and duration of sickness benefit [69]. No significant differences were found between workers in the intervention and usual care comparison group [69]. Another study evaluated the impact of flower-growing farmers’ adopting international standards to regulate product quality and production methods on labour, environmental, and social outcomes related to the production of flowers [67]. The study found overall self-reported improvements in worker and family welfare but also complaints of headaches, chest pain, skin rashes, and eye problems experienced by workers exposed to chemical agents, despite their access to and use of protective equipment [67]. Mainly preoccupied with process outcomes, the studies assessing the impact of the provision of health insurance plans on informal or precariously employed workers, reported on enrolment rates [73,76], health service utilization, and out-of-pocket payments [73], without focusing on specific health improvements as a result of having access to health insurance plans. Similarly, the study evaluating the likelihood of the Better Work program to improve working conditions in the apparel business reported on outcomes such as access to medication and workers’ perceptions of the quality of health clinics provided by the employer [74], but not on health outcomes themselves. The last study focused on a subjective measure of well-being, more specifically permanent and temporary workers’ job satisfaction in relation to the adoption of employment protection legislation for permanent workers [68]. It reported that permanent workers were satisfied with employment protection legislation but less satisfied with restrictions to temporary employment, possibly because, as hypothesized by the authors, having fewer temporary workers could diminish their advantages as permanent workers [68]. Similarly, temporary workers were satisfied with employment protection legislation adopted to protect permanent workers possibly because they anticipated benefiting from higher protection once securing a permanent contract. However, they were not satisfied with restrictions to temporary employment given that, in the short-term, such restrictions prevented them from getting their contracts renewed [68].

To make it easier to contextualize the 11 initiatives in relation to their potential to also affect workers’ employment conditions, we include a brief review of the PE-related outcomes evaluated for each initiative. To do this, we categorize these outcomes using the three dimensions of PE—employment insecurity, income inadequacy, and lack of rights and protection in the employment relation—discussed in the 2020 Kreshpaj et al. systematic review of PE operationalizations [10]. Overall, five [67,70,73,74,76] of the eight studies [67,68,70,72,73,74,75,76] addressing employment conditions included improvements in PE-related outcomes. Out of three studies concerned with changes in employment insecurity outcomes [67,70,75], two documented improvements [67,70]. All three studies examining changes in workers’ income levels found some evidence that the respective initiatives were successful in addressing income inadequacy [67,70,74], and of the four studies preoccupied with the lack of rights and protection in the employment relation [70,73,75,76], three found positive changes. Two found improvements in workers’ access to social and health benefits [73,76] and one found increased levels of union membership and collective agreement coverage, increased access to social insurance benefits, protection against customer abuse, and increased access to training and upward mobility for workers in large cleaning companies [70].

### 3.4. Facilitators and Barriers 

Several barriers and facilitators corresponding to both macro- and meso-levels have been identified across the studies as potentially contributing to the successful implementation of initiatives (Table 5). The lack of national standards to regulate employment and working conditions and the lack of enforcement of such standards [67], the extent of the informal economy and the large number of informal workers in some countries [77], along with the presence of market forces sustaining demand for an informal economy and informal workers [70] were among the macro-level barriers mentioned. Conversely, general government support [76], the regulation and enforcement of core labour standards at the national level [75], and collaborations among governments, employers and workers organizations, non-governmental organizations (NGOs), the ILO, and unions [70,72,77] were among the macro-level supports discussed. At the meso-level, some of the barriers mentioned included low density of unions and other forms of organized labour movements [70,75], inadequate or insufficient resources to enforce labour standards within organizations [74], low compliance with minimum labour standards and occupational health and safety requirements in low regulated industries [75], addressing only some but not all problems identified by workers [69], and underreporting of worker rights violations due to stigma associated with certain industries [75]. Several facilitators mentioned at the meso-level included the adoption of a safety culture by organizations [71], public pressure by consumers and investors to improve employment and working conditions [74], detailed implementation and evaluation planning [72], involvement of local stakeholders [71] and existing human and organizational networks [73,77], and the use of participative approaches involving all workers [71,77].

### 3.5. Quality Assessment

The results of the MMAT quality assessment, including the detailed rating for each criterion, are displayed in Table 6. Overall, with a few exceptions [67,69,71,73], most studies contained only limited descriptions of their research methods and evaluation process, thus making it challenging to find the details necessary to appraise each criterion included in the quality appraisal tool. For instance, for five studies [68,70,74,75,77], we selected the ‘Can’t tell’ response category at least once, and up to three times per study, given that there were insufficient methodological details reported to allow us to answer ‘Yes’ or ‘No’ to the quality assessment questions. 

In addition to study limitations regarding the reporting of methods and evaluation design, there were several methodological limitations across the included studies, as detailed in Table 6. There was variation in the overall quality of the 11 studies. All studies except for one [70] stated their research questions clearly, and most studies, except for three [68,70,74] collected data needed to address the research questions. Two [67,71] of the five qualitative studies met all seven quality criteria included in the MMAT, two [75,77] met at least four, and one [70] met none. The one randomized controlled trial [69] fulfilled six of the seven quality criteria and the non-randomized study [73] fulfilled all of them. Two [68,74] of the three quantitative descriptive studies fulfilled three quality criteria and one [76] fulfilled five. The one study using mixed methods [72] fulfilled four of the seven quality criteria. Although the study using mixed methods [72] was not labelled as such by the authors, based on its approach and combination of quantitative and qualitative methodology we assessed it using the MMAT criteria for mixed-methods studies. Contrary, given its exclusive use of qualitative methods, one study [75] that was identified as using mixed-methods by its author was assessed using the MMAT criteria for qualitative studies.

We did not exclude any studies based on the results of the critical appraisal, given the small number of studies found and the MMAT recommendations [63]. However, to support interpretation of findings and ensure that the evidence synthesised is considered in light of the methodological limitations and the risk of bias for included studies, we calculated a quality appraisal rating for each study. We recognize that the computation of an overall score is discouraged by the authors of the MMAT, who suggest as an alternative the detailed presentation of ratings for each criterion [63], as displayed in Table 6. To calculate the rating, we used the number of ‘Yes’ responses to the quality assessment questions, including the two screening questions, categorizing studies with 6–7 ‘Yes’ answers as high quality, studies with 3–5 ‘Yes’ answers as medium quality and studies with 0–2 ‘Yes’ answers as low quality (Table 1). Based on this categorization, four studies were determined to be high quality [67,69,71,73], five were medium quality [68,72,74,75,77], and one was low quality [70]. We included this rating in Table 4 along with the outcomes reported for each study so they can be considered in conjunction with each other. 

Given that the initiatives and outcomes reviewed in each of the 11 studies were distinct and there was no replication across the studies, in addition to assessing the quality and risk of bias for each individual study, we did not rate the overall body of evidence for any given outcome.

## 4. Discussion

Our literature review confirmed that, although there is a large body of evidence concerned with employment conditions in general and their impact on health and well-being [9], there is scarce evidence related to initiatives that can address PE specifically and its effects on the health and well-being of workers and their families [78]. Furthermore, the impact of initiatives on general employment conditions is seldomly evaluated [9]. Although we screened over eight thousand records, we found only 11 studies addressing PE that evaluated and reported the health and well-being effects of the initiatives. While the 11 initiatives are an encouraging start for stakeholders interested in addressing PE, the limited number of relevant initiatives found is disappointing. This finding clearly conveys the message of an existing gap regarding initiatives that have been both implemented and evaluated that were purposefully designed to address PE or its impact on the health and well-being of workers. This systematic review allowed us to confirm this gap and prepare a set of concrete recommendations on ways to address it. 

The range of initiatives found was quite diverse, encompassing interventions implemented in a variety of economic and political contexts across 22 countries belonging to four WHO regions and four continents, representing a combination of low, lower-middle, upper-middle, and high-income countries. An interesting finding is that none of the 11 studies examined countries located in the Americas, although the discussion on employment related aspects has been lively in both North and South America. This could reflect a weak interest among researchers in the Americas to evaluate PE initiatives related to health. An alternative explanation is that stakeholders implementing initiatives to address PE (i) are not preoccupied with the health and well-being effects of such initiatives, (ii) are not interested in or do not have capacity to conduct evaluations, or (iii) are not inviting researchers to conduct such evaluations. Another explanation could be that initiatives addressing PE in the Americas are focused on unemployment, which was one of our exclusion criteria, or that they are framed mainly as NSE.

The initiatives reviewed in each of the 11 studies were distinct and there was no replication across the studies. The initiatives targeted different worker sub-groups, including informal workers, and different economic sectors, including agriculture, textile industry, construction, sex industry, and domestic services. These sectors are commonly recognized for their limited enforcement of labour legislation, precarious employment conditions, high proportion of informal workers, and, except for the construction industry, high proportion of migrant women [50,79]. Although several of the described initiatives were implemented at the national or individual levels, most initiatives were implemented at the organizational level. Many initiatives showed the potential to improve the health and well-being of workers, but not all showed concern for improving aspects of workers’ employment conditions, such as employment instability, income inadequacy, or lack of rights and protections. The challenge of integrating health and labour concerns could reflect disciplinary and methodological differences between researchers in these two fields, typically working in silos despite ongoing efforts to increase collaboration across disciplines [80].

This mixture of initiatives is not surprising given that PE takes many shapes and forms across the world and that there are no consistent approaches or standardized ways to address the multitude of PE-related problems. The diversity of initiatives is paralleled by a diversity of approaches to implementation, evaluation, and reporting of findings, reflecting an extensive range of study designs and evaluation methods available. The wide range of economic and political contexts in which these initiatives were implemented, the diverse population sub-groups and economic sectors targeted, along with the myriad of implementation approaches and evaluation designs make it difficult to compare outcomes across initiatives, generalize findings, and issue recommendations. 

While reviewing the 11 initiatives and their evaluation processes, it became clear that the evaluation approaches are very diverse, and that the evaluation component and the outcomes measured are often described with less detail than the initiative itself. This makes it difficult to assess the evaluation and to reach conclusions about the reliability of findings and reproducibility of initiatives in other contexts. Moreover, lack of detail about the process undertaken to evaluate the impact of initiatives also prevents a clear understanding of weaknesses in the implementation or evaluation so that these can be avoided in the future. Additionally, since most evaluations used across the 11 studies relied mostly on process indicators, it is not clear if, in the short- or long-term, the improvements in process indicators were accompanied by improvements in outcome indicators. Given that, with a few exceptions, the specific impact of initiatives on population sub-groups according to socio-demographic characteristics such as age, gender, education, or ethnicity was not reported in the studies, we were not able to assess population sub-group impacts. 

### 4.1. Practice Implications

Overall, all initiatives described in the 11 studies showed the potential to improve the health and well-being of workers and could be considered for implementation in a range of contexts. However, given the diversity of both the initiatives and the approaches used to evaluate them, stakeholders interested in addressing PE should carefully consider the population sub-groups and economic sectors they wish to target, the dimensions of PE and health aspects they are interested in improving, as well as the available resources and implementation supports. 

Furthermore, to facilitate uptake of planned initiatives and ensure their thorough evaluation, organizations interested in adopting and evaluating initiatives to address PE should seek collaborations with implementation science researchers and other researchers, as well as program planning and evaluation specialists.

### 4.2. Research Implications

It is essential to enhance research efforts to ensure that all initiatives addressing PE undergo planned and thorough evaluations to examine their impact and the specific effects they have on the health and well-being of populations. Such evaluations should include collection of baseline indicators before initiatives are implemented, collection and use of tested indicators of PE [47,48], and use of robust research designs and methodologies, along with thorough documentation of the process, to ensure that others can tailor initiatives deemed successful to their own contexts. In addition, when planning and measuring the impact of initiatives, researchers should aim for a balance of process and outcome indicators.

Furthermore, given the multitude of detrimental effects that PE has on health, any study preoccupied with examining the impact of initiatives targeting PE should consider more than the impact of an intervention on PE prevalence. Researchers should consider evaluating and reporting the effects that interventions have on workers’ mental and physical health, in addition to overall well-being and occupational health and safety indicators [81]. Since interventions can affect groups differently, it is essential that evaluations allow for the disaggregation of data according to type of PE and socio-demographic factors such as age, gender, education, and ethnicity to facilitate a better understanding of the impact of such initiatives on population sub-groups. In addition, the unequal distribution of PE along socially created axes of disadvantage such as race, gender, education, income, class, immigration status, and citizenship [8,21,36,50,51,82] offers a strong justification for assessing the effects of employment-related initiatives on health equity. To enable the application of these considerations when assessing the risk of bias in individual studies and the overall strength of available evidence, these factors should also be accounted for in critical appraisal and grading tools. 

Given the heightened interest in the topic of PE and need for solutions triggered by the COVID-19 pandemic, having access to a vast repertoire of initiatives that have been tested in different contexts (e.g., different countries, income-levels, economies, population sub-groups, etc.) would facilitate the adoption of interventions that show robust potential to eliminate, reduce, or alleviate workers’ exposure to PE and improve their health and well-being outcomes. Once identified, publications describing such initiatives and the results of their evaluations could be shared more widely with interested audiences through centralized database systems, such as the ones managed by the ILO, Eurofound, or the WHO. Such repositories could be structured by characteristics such as initiative purpose, context, target population and industry, dimension of PE addressed, evaluation type, and specific outcomes to make it easier for interested stakeholders to find them. Of course, this would be a complex and costly endeavour that would require considerable effort and ongoing maintenance. However, we believe that the importance of the PE topic and the current global preoccupation with identifying solutions to the myriad of PE problems justify the allocation of resources towards this goal. The *Platform economy repository* hosted by the Eurofound, provides an example of such a database, with its focus on initiatives addressing the platform economy [83]. 

### 4.3. Policy Implications

When considering whether an initiative should be implemented at a macro-, meso-, or individual level, close consideration should be given to the nature and urgency of the specific PE-related problems faced, the economic and political context, the financial and human resources available, as well as the populations and economic sectors targeted. While in certain situations the implementation of policies and other macro-level initiatives could have a higher potential to facilitate practice changes and health improvements [84,85,86,87], in others, smaller-scale efforts in local settings or that target more specific problems or populations may have greater likelihood of success [87,88].

Limitations in the evaluations included in this review could be a symptom of inconsistent and insufficient allocation of funding towards evaluation efforts. To address this, local or national governments interested in the implementation and evaluation of initiatives to address PE should earmark consistent and sufficient funding for the evaluation component of any new initiative implemented. Stakeholders such as trade unions, workers’ and employers’ organizations, and insurance companies could advocate for policy makers to allocate long-term funding for hiring enough professionals to conduct robust evaluations and for collection and analysis of adequate indicators.

In addition to our policy recommendations relevant to research focused on PE initiatives and their evaluations, we include several recommendations regarding strategies to address PE. These recommendations build on the ILO 2020–2022 World Social Protection Report [23] as well as an earlier report containing suggestions for policies needed to address the multitude of insecurities affecting workers in NSE [12]. Key policy recommendations made by the ILO include (i) the improving of social protections and strengthening of collective bargaining, (ii) the adjusting of existing gaps in legislation to ensure the equal treatment of all workers no matter their employment arrangements, and (iii) the adoption of employment and social policies that help workers manage social risks such as unemployment, parental, and caring leaves [12]. Additionally, considering the numerous health and social protections gaps under intense scrutiny during the COVID-19 pandemic, the ILO suggests the adoption of recovery strategies that that are centred on the universal provision of health and social benefits [23]. Such strategies have the potential to decrease the vulnerability of workers affected by employment and income related insecurities [23]. 

### 4.4. Strengths and Limitations

This is the first systematic review conducted to identify, appraise, and synthesise implemented and evaluated initiatives that tackle PE and its impact on the health and well-being of workers. We hope that this review will function as a catalyst for additional research to expand the knowledge base on effective initiatives that tackle the myriad of PE-related problems and improve the health and well-being of workers. This much-needed research is especially important considering the continued increased prevalence of PE across the world and the intensification of aspects such as employment insecurity and income inadequacy.

The review adopted a multidimensional perspective when considering the construct of PE [10], which was reflected in the search strategy used, the definition of the inclusion/exclusion criteria, the consideration of the ways in which initiatives could address PE and the specific dimensions targeted, namely employment insecurity, income inadequacy, and worker rights. The review was conducted by an international team with combined expertise in occupational and social epidemiology, public health, nursing, social determinants of health, and health inequities. The size of the team allowed the full-text screening, data extraction, and quality appraisal to be completed independently by two reviewers, regardless of the large number of studies initially identified.

Despite developing an extensive search string to identify the many ways PE might be addressed in the research literature, the inconsistent use of PE definitions and the numerous terms and approaches used to refer to this construct may have resulted in our search and screening process missing potentially relevant studies or initiatives. Given the gap in synthesised knowledge about the outcomes of implemented evaluations addressing PE, there is a need for further systematic reviews that build on our work, for instance by searching more sources of the academic and grey literature and other languages that, given capacity limitations, we were not able to cover. It is quite plausible that there is information about relevant initiatives implemented and evaluated by workers’ and employers’ organizations, professional associations, and several other worker advocacy groups that is not yet shared widely with those who may benefit from it. Similarly, the languages in which such reports and publications are written could be different from the ones we were able to incorporate in this review. 

## 5. Conclusions

The prevalence of PE has increased in recent decades and aspects such as employment insecurity and income inadequacy have intensified during the COVID-19 pandemic. The exposure of workers to PE is linked to numerous health and well-being concerns and poses significant challenges to population health and health equity. In this manuscript we synthesise available evidence pertaining specifically to studies that have evaluated and reported the health and well-being outcomes of initiatives addressing PE, as found through a systematic review. This information could support policy makers, workers’ and employers’ organizations, researchers, and other relevant stakeholders in their efforts to improve the health and well-being of workers and tackle PE. We used the PRISMA framework to guide the review and identified 11 relevant initiatives through searches in PubMed, Scopus, Web of Science, and three sources of grey literature (published from January 2000 to May 2021). In addition to confirming a scarcity of knowledge regarding initiatives to address PE and its effects on the health and well-being of workers and their families, this review also identified that the impact of such initiatives is evaluated seldomly. 

We found very few evaluated interventions addressing PE and its impact on the health and well-being of workers globally. Ten out of 11 initiatives were not purposefully designed to address PE in general, nor specific aspects of it, such as employment instability, income inadequacy, or lack of rights and protection. Seven out of 11 initiatives evaluated outcomes related to the occupational health and safety of precariously employed workers and six out of 11 evaluated worker health and well-being outcomes, two of which also included a focus on workers’ families. Most initiatives showed the potential to improve the health and well-being of workers, although the evaluation component and the outcomes measured were often described with less detail than the initiative itself. Given the heterogeneity of the 11 initiatives with regard to study design, sample size, implementation, evaluation, economic and political contexts, and target population, we found insufficient evidence to compare outcomes across types of initiatives, generalize findings, or make specific recommendations for the adoption of initiatives. It is evident that further research is necessary to thoroughly evaluate the impact of initiatives addressing PE, understand the specific effects that such initiatives have on the health and well-being of populations, and share details of the findings to support the implementation of promising initiatives into practice.

## Figures and Tables

**Table 1:** Characteristics of the included studies.

Number of Studies Included		11
Continents represented by the countries examined	Africa	1
	Asia	6
	Europe	3
	Oceania	1
Study design *	Qualitative studies	5
	Randomized controlled trials	1
	Non-randomized controlled trials	1
	Quantitative descriptive studies	3
	Mixed methods studies	1
Targeted economic sector (ISIC Rev 4) **^,^ °	Agriculture, Forestry, and Fishing	3
	Manufacturing	4
	Construction	2
	Hotels and restaurants	1
	Transportation and storage	1
	Activities of households as employers; undifferentiated goods- and services-producing activities of households for own use	2
	Not elsewhere classified	1
	All economic sectors	2
Initiative being purposefully designed to address precarious employment	No	10
	Yes	1
Dimensions of PE potentially impacted °	Employment insecurity	4
	Lack of rights and protection in the employment relation	5
	Income inadequacy	4
Health and well-being outcomes evaluated °	Occupational health and safety	7
	Worker and/or family health and well-being	6
Quality appraisal rating ***	Low quality (0 to 2)	1
	Medium quality (3 to 5)	6
	High quality (6 to 7)	4

* This categorization of study design uses the categories included in the Mixed Methods Appraisal Tool (MMAT), 2018 version. ** https://ilostat.ilo.org/resources/concepts-and-definitions/classification-economic-activities/ (accessed on 15 December 2021); ° The sum could be more than 11 given that several studies targeted several economic sectors, evaluated several outcomes, and could have impacted several PE dimensions. *** Quality appraisal rating interpretation: To calculate the rating, we used the number of ‘Yes’ responses to the quality assessment questions included in the MMAT 2018 version, including the two screening questions; Low quality (0–2 ‘Yes’ answers), Medium quality (3–5 ‘Yes’ answers), and High quality (6–7 ‘Yes’ answers).

**Table 2:** Countries examined, type of evidence, study design, targeted economic sector, population sub-groups, and study objectives.

Study Author(s) Publication Year	Countries Examined	Type of Evidence	Study Design	Targeted Economic Sector and Population Sub-Groups	Study Objectives
Davies R., 2000 [67]	Zimbabwe	ILO institutional report	Qualitative study (Case study)	Agriculture Formal and informal flower-growing workers	To examine the impact of international labelling standards adopted by the flower-growing farmers in Zimbabwe on employment, income, and working conditions.
Manothum, A. et al., 2010 [71]	Thailand	Academic journal article	Qualitative study (Participatory action research)	Ceramic workers, plastic weavers, blanket makers, and pandanus weavers Informal sector workers	To evaluate the outcomes of a participatory approach used to promote OHS, based on informal sector workers’ (a) knowledge, attitudes, and behaviours in OHS, (b) work practice improvements, and (c) working conditions improvements.
Salvatori, A., 2010 [68]	13 OECD countries: Austria, Belgium, Germany, Denmark, Spain, Finland, France, UK, Greece, Ireland, Italy, Netherlands, Portugal	Academic journal article	Quantitative descriptive study (Longitudinal panel surveys)	No specific sector Permanent and temporary workers affected by employment protection legislation	To study the effects of employment protection legislation adopted for permanent workers and of restrictions on the use of temporary employment on individual workers’ wellbeing.
Kawakami, T. et al., 2011 [77]	Cambodia	Academic journal article	Qualitative study (Participatory action research)	Domestic service sector, small construction sites, rural farms Informal workers	To examine the impact of a participatory approach and use of participatory training methodologies on safety and health in informal workplaces.
Vermeulen, S.J. et al., 2011 [69]	Netherlands	Academic journal article	Randomized controlled trial	No specific sector Temporary and unemployed agency workers sick-listed due to musculoskeletal disorders	To evaluate the effectiveness of a participatory return-to-work program to facilitate work resumption and reduce work disability for unemployed workers and temporary agency workers, who are off on sick leave due to musculoskeletal disorders.
Bowman, J.R et al., 2014 [70]	Sweden	Academic journal article	Qualitative study (Case study)	Domestic service sector Cleaning workers, both formal and informal	To describe the impact of a governmental tax policy that subsidizes the hiring of domestic cleaning workers on the creation of better working conditions for them.
Brown, D. et al., 2014 [74]	Vietnam	Book chapter	Quantitative descriptive study (Case study)	Textile industry Factory workers	To conduct a preliminary assessment of the impact of the Better Work Vietnam program on compliance with national and international labour regulations and on factory and worker well-being. Given its focus on compliance and the use of non-primary evidence, the evaluation of the Better Factories Cambodia program, also included in this chapter, is not part of our analysis.
Orchiston, A., 2016 [75]	Australia	Academic journal article	Qualitative study (Case study)	Sex industry Brothel-based sex workers	To study the relationship between sex workers’ working conditions and two regulatory models governing sex work (decriminalisation and licencing).
Rothboeck, S. et al., 2018 [72]	India	Academic journal article	Mixed methods	Agriculture, Healthcare Gems and jewellery; Domestic sector Workers in sectors with high informality	To examine the impact of the ‘Recognition of Prior Learning’ initiative on income opportunities, occupational safety, social status, and openness to further learning.
Khan, J.A.M. et al., 2020 [73]	Bangladesh	Academic journal article	Non-randomized study (Quasi-experimental)	Rickshaw pullers, shopkeepers, restaurant workers, day laborers, factory workers and transport workers in rural areas Informal workers	To estimate the effect of a community-based health insurance scheme on the magnitude of out-of-pocket healthcare payments made by informal workers and their dependents for health services.
Si, W., 2021 [76]	China	Academic journal article	Quantitative descriptive study (Cross-sectional)	No specific sector Urban population not covered by employment-based health insurance.	To estimate the effects of a national public health insurance program on health and on various labour market outcomes such as long-term and limited duration employment, and self-employment.

The blue colour is used to denote the targeted economic sector.

**Table 3:** Description of initiatives, ways in which they could impact PE, and design and data collection approaches used to evaluate them.

Study Author(s) Year of Publication	Implemented Initiatives Initiative Being Purposefully Designed to Address Precarious Employment	Ways in Which the Initiative Could Impact PE Specific Dimension(s) of PE Potentially Impacted	Initiative Level	Design and Data Collection Approaches Used to Evaluate Initiatives
Davies, R., 2000 [67]	**Flower-growing farmers’ adoption of international standards regulating flower quality and producing methods.** Such standards regulate labour, environmental, and social aspects related to the production of flowers. The labour aspects regulated involve collective bargaining, employment security, equal treatment, wage setting processes, health and safety practices, and banning of child labour. The environmental aspects control the use of crop protection agents and fertilizers, energy utilized, disposal of toxic agents, and waste production, while the social aspects controlled include pay levels, living and working facilities, and respecting human rights. No	Eliminate, reduce, or mitigate workers’ exposure to informal employment or PE and their effect on workers’ health and well-being. Employment insecurity	Meso level	Field interviews and surveys conducted with 5 farmers and 34 workers at five farms that adopted labelling standards. Workers were asked to compare health and safety practices and job characteristics with those at previous farms they worked at that did not adapt the standards. Additional information collected from collective bargaining agreements, statistical data, and personal communication with experts in the field.
Manothum, A. et al., 2010 [71]	**The adoption of aparticipatory process to involve informal workers in addressing and solving occupational health and safety risks** to minimize their health effects and prevent them in the long-term. The initiative included capacity building, risk analysis, problem prevention and solving, and monitoring and communication; it was developed in partnership with local networks, non-governmental organizations, governmental officials, and informal worker leaders. No	Mitigate the effect of informal employment on workers’ health and well-being.	Meso level	Evaluation of data collected before and after the implementation of the participatory process approach measuring: (1) knowledge, attitudes, and behaviours, using a questionnaire developed by the Department of Labor in Thailand; (2) work practice improvements, using an ILO-developed checklist; and (3) heat and lighting, using industrial hygiene instruments.
Salvatori, A., 2010 [68]	**Adoption of employment protection legislation** for permanent workers and of restrictions on the use of temporary employment to protect workers with permanent contracts. Differences in the type of legislation adopted across the 13 countries and 7 years analysed. Yes	Limit increases in prevalence of PE. Employment insecurity Lack of rights and protection in the employment relation.	Meso level	Seven waves (1994–2001) of the European Community Household Panel used to collect data on a subjective measure of well-being (job satisfaction) from a large sample of temporary and permanent employees in 13 OECD countries. The exposure variable, employment legislation, measured with two OECD aggregated indicators, an employment protection legislation index and an index assessing restrictions on the use of temporary employment. The outcome of interest measured for both permanent and temporary workers and compared across 13 countries and 7 years, taking advantage of between country and yearly variation in employment protection legislation.
Kawakami, T. et al., 2011 [77]	A **participatory training program** developed collaboratively by the government, employers, NGOs, and workers and delivered by safety and health trainers. Key program steps: (i) the identification of existing health and safety practices; (ii) the development of new participatory training programmes based on ILO training programmes; and (iii) the training of safety and health trainers using a train the trainer model. No	Mitigate the effect of informal employment on workers’ health and well-being.	Micro level	Workplace visits conducted to collect process and outcome indicators such as number of people trained, types of training tools developed, and types of improvements implemented after the adoption of the initiative.
Vermeulen, S.J. et al., 2011 [69]	A **participatory return-to-work program** was developed to make return-to-work for unemployed workers and temporary agency workers more effective. Typically, these workers do not have a workplace or an employer to return to. The sick-listed worker, a labor expert from a social security agency, an independent return-to-work coordinator, and a rehabilitation agency sought solutions to barriers related to physical environments, job and role demands, work experience requirements, commuting, and other factors. This included the finding of a suitable therapeutic workplace for workers to join after their sick leave. No	Promote workers’ return to work after illness or injury in a way that mitigates their PE.	Micro level	Sick-listed workers were randomly allocated to the participatory return-to-work program or to usual care, consisting of supportive income and rehabilitation support and guidance. Data were collected from a social security agency database and self-report questionnaires completed by workers. Outcomes were measured at baseline, 3, 6, 9 and 12 months. Duration of sickness benefit was defined as the length of time from random allocation to the program until stopping the sickness benefit for at least 28 days. Functional status and general health were assessed through the MOS 36-item short-form health survey (SF-36). Musculoskeletal pain intensity was evaluated using the Von Korff questionnaire.
Bowman, J.R et al., 2014 [70]	Adoption of a **tax policy that provides a tax break to households that hire domestic cleaning workers** to help with household activities such as cleaning, cooking, childcare, and gardening/yard work. The cash refund, equivalent to 50% of the hiring costs, subsidizes the cost of hiring domestic workers and reduces households’ costs for hiring them. The purpose of the tax break was twofold: to generate entry-level jobs and reduce undeclared labour in the domestic cleaning sector by making domestic labour services affordable for households without them having to bend the rules. No	Eliminate, reduce, or mitigate workers’ exposure to informal employment or PE and their effect on workers’ health and well-being. Employment insecurity Income inadequacy Lack of rights and protection in the employment relation	Macro level	Semi-structured interviews conducted with cleaning workers’ union leaders, employer organizations, a labour union and an advocacy organization representing undocumented workers, employers and employees from a large cleaning firm, journalists, and party politicians to assess perceptions about the job conditions of domestic service sector cleaners after the introduction of the tax policy as compared with their conditions before.
Brown, D. et al., 2014 [74]	The adoption of **Better Work Vietnam program**, aimed at improving employment and working conditions in the apparel business. The program is based on the Better Work global program model of monitoring compliance with existing labour regulations through partnerships between unions, factory management representatives, government, and market stakeholders. The program consists of mandated adherence to national and international labour standards, monitored by the ILO through a 200-question assessment instrument, along with the mandatory formation of a performance improvement working committee, and the performance of assessment visits by official ILO monitors hired locally. No	Eliminate, reduce, or mitigate workers’ exposure to PE and its effects on workers’ health and well-being. Income inadequacy	Meso level	Pre- and post- implementation data on worker demographics, employment and working conditions (wages, relationship with management, communication), and factory level information regarding program adoption collected through worker surveys. The exposure measures used were length of time since the formation of the performance improvement working committee and length of time since the first assessment visit by official monitors.
Orchiston, A., 2016 [75]	**Adoption of regulatory frameworks, either decriminalisation or licencing, to govern sex work**. The decriminalisation of sex work framework analysed included the repeal of most criminal laws concerning commercial sex activities, the categorization of brothels or other establishments where sex work takes place as lawful, and the subjecting of brothels to the same laws governing other legal commercial businesses. The licencing system framework reviewed required that brothels must be licenced and must adhere to strict licencing requirements overseen by a dedicated government agency. In addition, establishment owners and managers must undergo criminal record checks. No	Eliminate, reduce, or mitigate workers’ exposure to PE and its effects on workers’ health and well-being. Employment insecurity Income inadequacy Lack of rights and protection in the employment relation	Macro level	30 semi-structured interviews with individuals involved in the sex industry (sex workers, brothel managers, key professionals) to evaluate perceptions on brothel sector working conditions and workplace rights, labour practices, and assess various indicators of employment precariousness. Interview data triangulated through content analysis of 54 weblogs. Document analysis of written contracts, codes of conduct, internal communication and signage also performed. Outcomes compared and contrasted across the two legal frameworks reviewed.
Rothboeck, S. et al., 2018 [72]	A **recognition of prior informal learning initiative** meant to promote inclusive skill development and increase employability through (i) facilitating easier access to technical and vocational education and training for informal workers and (ii) increasing flexibility in obtaining skill recognition and certification. The initiative was piloted in four different industries and the government of India, ILO, and representatives in four economic sectors collaborated for the implementation of the pilots. No	Eliminate, reduce, or mitigate workers’ exposure to informal employment or PE and their effect on workers’ health and well-being. Income inadequacy	Meso level	A baseline survey, two sets of follow-up surveys, field visits, and focus discussion groups conducted to assess the design and implementation of the four pilots and their impact on the targeted workers. In total, 3150 individuals recruited. The assessment of worker outcomes before and after (6 and 18 months, respectively) implementation of the pilots.
Khan, J.A.M. et al., 2020 [73]	A **pilot community-based health insurance scheme** implemented within seven administrative units belonging to a worker cooperative in a rural area. The scheme consisted of a package of health and non-health benefits offered to informal workers and family members in exchange for an ongoing membership fee and low co-payments upon accessing services. No	Reduce and mitigate the effect of informal employment on workers’ health and well-being. Lack of rights and protection in the employment relation	Meso level	Structured face-to-face interviews administered to 1292 households (646 insured and 646 uninsured) to estimate differences in out-of-pocket healthcare payments between insured and non-insured households in the 3 months before the survey. Out-of-pocket payments consisted of medical fees, charges for public hospital care, co-payments for health insurance, and the costs for medicine purchases, medical appliances, and diagnostic tests.
Si, W., 2021 [76]	A **voluntary national public health insurance program** implemented to offer coverage to residents in urban regions who do not benefit from employment-based insurance (e.g., the elderly, children, college students, unemployed workers, self-employed, and informally employed). Participation premium fees subsidized to a high degree by the government, but individuals must partially contribute to the premium fees. No	Reduce and mitigate the effect of PE employment on workers’ health and well-being. Lack of rights and protection in the employment relation	Macro level	Panel data from the China Health and Nutrition Survey, the 2004, 2006, 2009, and 2011 waves used to assess enrolment rates of working age individuals in the national health insurance program, along with several employment mobility indicators. A comparison of indicators across cities that adopted and those that did not yet adopt the program was performed, facilitated by a gradual implementation of the program within the country.

The green colour is used to indicate if the initiatives described were purposefully designed to address precarious employment. The purple colour is used to indicate the ways in which the initiative could impact PE.

**Table 4:** Brief description of initiatives, health and well-being outcomes, and PE outcomes evaluated.

Study Author(s) Year of Publication	Implemented Initiative Brief Review of Each Implemented Initiative	Health and Well-Being Outcomes Evaluated Divided into 2 Categories: Occupational Health and Safety and Worker and Family Health and Well-Being	PE Outcomes Evaluated Divided into 3 Categories: Employment Insecurity, Income Inadequacy, and Workplace Rights	Quality Appraisal Rating *
Davies, R., 2000 [67]	Flower-growing farmers’ adoption of international standards regulating flower quality and producing methods.	**Occupational health and safety**—Improvements in health maintenance practices such as workers being sent for regular blood tests and check-ups through health clinics. **Worker and family health and well-being**—Overall self-reported improvements in worker and family welfare. Despite the provision and use of protective equipment, workers closely exposed to chemical agents reported headaches, chest pain, skin rashes, and eye problems.	**Employment insecurity**—An increase in the number of permanent workers, including female permanent workers, and the replacement of verbal agreements with written employment contracts. **Income inadequacy**—Higher wages when compared with the wages workers gained while working at other farms.	High
Manothum, A. et al., 2010 [71]	The adoption of a participatory process to involve informal workers in addressing and solving occupational health and safety risks.	**Occupational health and safety**—Increased use of PPE equipment; increases in workers’ knowledge, attitudes, and behaviours with regard to work safety practices; increased understanding of job safety; and working conditions improvements including reduced exposure to heat and increased lighting to meet governmental standards.	No PE outcomes evaluated.	High
Salvatori, A., 2010 [68]	Adoption of employment protection legislation.	**Worker health and well-being**—For permanent workers, job satisfaction had a positive relationship with employment protection legislation and a negative relationship with restrictions to temporary employment. For temporary workers, job satisfaction had a positive relationship with employment protection legislation covering permanent workers and a negative relationship with increased restrictions to temporary employment.	The initiative addressed PE, but no PE outcomes were evaluated.	Medium
Kawakami, T. et al., 2011 [77]	A participatory health and safety training program.	**Occupational health and safety**—Increased access to action checklists and training tools on the handling of materials and tools, machine operation safety, working at heights, work ergonomics, physical environment hazards (pesticide handling, lighting, ventilation, use of PPE) and welfare facilities (drinking water, resting facilities, toilets). A total of 5111 workers trained on various strategies to improve safety and work environment conditions.	No PE outcomes evaluated.	Medium
Vermeulen, S.J. et al., 2011 [69]	A participatory return-to-work program to facilitate work resumption and reduce work disability, among unemployed workers and temporary agency workers.	**Worker health and well-being**—No significant differences found between workers in the intervention and usual care comparison groups with regard to functional status, pain intensity, perceived health, and duration of sickness benefit.	No PE outcomes evaluated.	High
Bowman, J.R et al., 2014 [70]	Adoption of a tax policy that provides a tax break to households that hire domestic cleaning workers.	**Occupational health and safety**—Workers benefited from training or information provided by domestic cleaning companies with regard to ergonomics while conducting cleaning work, safety labels, and environmental certification of cleaning products.	**Employment insecurity**—Improvements in job security given that workers moved from an informal job or from being self-employed to being formally employed by a documented company created as a result of increased demand for cleaning services after the introduction of the tax. **Income inadequacy**—Wage increases for workers belonging to unionized cleaning companies. **Workplace rights**—Improvements in union membership and collective agreement coverage, increased access to social insurance benefits, protection against customer abuse, and increased access to training and upward mobility for workers in large cleaning companies.	Low
Brown, D. et al., 2014 [74]	The adoption of Better Work Vietnam program, aimed at improving employment and working conditions in the in the apparel business.	**Occupational health and safety**—No significant relationship was observed between the two exposure measures and factors such as exposure to extreme temperatures, concerns about dangerous equipment, and accidents and injuries. The length of time since first assessment visit by official monitors was associated with perceptions of higher quality of the health clinics offered by the factory. Worker health and well-being—The length of time since the performance improvement working committee was created was associated with increased access to free medicine and with more concerns about the quality of the health clinics provided by the factory.	**Income inadequacy**—Small and non-statistically significant wage improvements.	Medium
Orchiston, A., 2016 [75]	Adoption of regulatory frameworks, either decriminalisation or licencing, to govern sex work.	**Occupational health and safety**—The decriminalisation approach was comparatively less effective in promoting adherence to occupational health and safety legislation than the licencing framework, which incorporates mandatory safety obligations as part of its licencing requirements. However, effective supervision is needed for any of these models to be effective.	**Employment insecurity**—Neither of the two regulatory models studied was successful in addressing the problems of bogus self-employment and dismissal. **Workplace rights** -Licencing promoted stronger compliance with occupational health and safety law than decriminalisation but inefficient supervision reduced the effect on PE for both. - Neither of the two regulatory models studied was successful in enforcing a minimum standard of fair working conditions.	Medium
Rothboeck, S. et al., 2018 [72]	An initiative to recognize workers’ prior informal learning.	**Occupational health and safety**—Increased awareness of occupational health and safety issues and application of safety practices across all four industry sectors examined, with the most improvements observed in the agriculture and gems/jewellery sectors.	**Income inadequacy**—No significant wage improvements. Although 4% of workers reported a positive effect on their income at both 6 and 18 months and 26% of workers reported some improvements either at 6 or 18 months, the improvements were short-term only and potentially linked to other factors.	Medium
Khan, J.A.M. et al., 2020 [73]	A pilot community-based health insurance scheme.	**Worker and family health and well-being**—Insured households, when compared to uninsured ones were 1.43% more likely to utilize medically trained professionals and their overall out-of-pocket payments for health services provided by medically trained professionals were 6.4% lower. No significant differences found in the overall out-of-pocket payments for health services provided by other types of healthcare providers, both trained and untrained. An individual’s asset quintile, residential location, illness type and inpatient care utilization had a significantly positive effect on out-of-pocket payments. Being unmarried had a significantly negative effect on out-of-pocket payments.	**Workplace rights**—Improvements in access to social and health benefits.	High
Si, W., 2021 [76]	A voluntary national public health insurance program.	**Worker health and well-being**—A health improvement effect was suggested by the finding that previously unhealthy individuals had an increased probability of being in fixed-term contracts and in self-employment after enrolment in the insurance program. Enrolment rates in the national health insurance program were: (i) similar among self-reported healthy and unhealthy groups, (ii) slightly higher among men than among women, and (iii) higher among individuals who were not working previously or who worked in the informal sector than among those working in the formal sector.	**Workplace rights**—Improvements in access to social and health benefits.	Medium

* Quality appraisal rating interpretation: To calculate the rating, we used the number of ‘Yes’ responses to the quality assessment questions included in the MMAT 2018 version, including the two screening questions. Low quality (0–2 ‘Yes’ answers), Medium quality (3–5 ‘Yes’ answers), and High quality (6–7 ‘Yes’ answers).

**Table 5:** Common macro- and meso-level barriers and facilitators to the successful implementation of initiatives.

	Barriers
Macro	The extent of the informal economy and the large number of informal workers in some countries are difficult to tackle unless structural, high-level solutions are considered [77].
	Market forces sustaining demand for an informal economy and informal workers make it difficult to reduce the informal economy [70].
	Lack of national standards to regulate certain employment and working conditions, lack of enforcement of such standards, and lack of local inspectorates to perform inspections when international standards are adopted [67].
	Low, seasonal, and inconsistent enrolment in large health insurance schemes affects their viability [73].
**Meso**	Low density of unions and other forms of organized labour movements within some industries [70,75].
	Inadequate or insufficient resources to enforce labour standards within organizations [74].
	Low compliance with minimum labour standards and occupational health and safety requirements in less regulated industries strongly influenced by market forces [75].
	Difficulties encountered with the piloting of initiatives due to insufficient knowledge about their nature and reluctance by both employers and workers to participate [72].
	Lack of accurate baseline data [72].
	Acknowledging and/or addressing only some of the identified problems affecting workers [69].
	Stigma associated with certain industries (e.g., sex work) prevents workers from filing complaints or making use of legal processes to help them challenge situations in which their rights are not met [75].
	**Facilitators**
Macro	General government support [76].
	The regulation and enforcement of core labour standards at the national level [75].
	Collaboration between government, governmental ministries, employers and workers organizations, NGOs [77], collaboration between government, economic sectors, and the ILO [72], collaboration with unions [70].
	Inclusion of informal economy workplaces in the national occupational health and safety agenda [77].
Meso	The adoption of a safety culture by organizations [71].
	Public disclosure of monitoring results and public pressure by consumers and investors to improve employment and working conditions [74].
	Efforts to support implementation of initiatives [72,77], such as detailed planning to facilitate enrolment, data collection, and built-in evaluation processes [72] as well as follow-up visits [77].
	Involvement of local stakeholders [71].
	Learning from and building on successful strategies already tested locally (by other employers and workers) [71,77].
	Participative approaches involving all workers [71,77].
	Existing preoccupation of employers with improving employment and working conditions even before the implementation of related initiatives [67].
	Involving competent and independent professionals in occupational health and safety initiatives [69].
	The use of human networks to reach informal workers who are typically not easily accessed by government organizations because of their informality [77].
	The use of existing networks of worker cooperatives, the offering of complementary non-health benefits, and the contracting of high-quality health services and professionals [73] and the subsidizing of the participation premium fees by the government [76] can increase the viability, quick expansion, and success of large insurance schemes.

**Table 6:** Risk of bias assessment of included studies using the Mixed Methods Appraisal Tool (MMAT).

Study Author(s) Year of Publication	Screening Questions		1. Qualitative Studies				
	S1. Are there clear research questions?	S2. Do the collected data allow to address the research questions?	1.1. Is the qualitative approach appropriate to answer the research question?	1.2. Are the qualitative data collection methods adequate to address the research question?	1.3. Are the findings adequately derived from the data?	1.4. Is the interpretation of results sufficiently substantiated by data?	1.5. Is there coherence between qualitative data sources, collection, analysis and interpretation?
Davies, R., 2000 [67]	Yes	Yes	Yes	Yes	Yes	Yes	Yes
Manothum, A. et al., 2010 [71]	Yes	Yes	Yes	Yes	Yes	Yes	Yes
Kawakami, T. et al., 2011 [77]	Yes	Yes	Yes	Yes	Can’t tell	Can’t tell	Can’t tell
Bowman, J.R et al., 2014 [70]	No	Can’t tell	No	Can’t tell	No	No	No
Orchiston, A., 2016 [75]	Yes	Yes	Yes	Yes	Yes	Can’t tell	Can’t tell
	**Screening Questions**		**2. Randomized Controlled Trials**				
	S1. Are there clear research questions?	S2. Do the collected data allow to address the research questions?	2.1. Is randomization appropriately performed?	2.2. Are the groups comparable at baseline?	2.3. Are there complete outcome data?	2.4. Are outcome assessors blinded to the intervention provided?	2.5 Did the participants adhere to the assigned intervention?
Vermeulen, S.J. et al., 2011 [69]	Yes	Yes	Yes	Yes	Yes	No	Yes
	**Screening Questions**		**3. Non-Randomized Studies**				
	S1. Are there clear research questions?	S2. Do the collected data allow to address the research questions?	3.1. Are the participants representative of the target population?	3.2. Are measurements appropriate regarding both the outcome and intervention (or exposure)?	3.3. Are there complete outcome data?	3.4. Are the confounders accounted for in the design and analysis?	3.5. During the study period, is the intervention administered (or exposure occurred) as intended?
Khan, J.A.M. et al., 2020 [73]	Yes	Yes	Yes	Yes	Yes	Yes	Yes
	**Screening Questions**		**4. Quantitative Descriptive Studies**				
	S1. Are there clear research questions?	S2. Do the collected data allow to address the research questions?	4.1. Is the sampling strategy relevant to address the research question?	4.2. Is the sample representative of the target population?	4.3. Are the measurements appropriate?	4.4. Is the risk of nonresponse bias low?	4.5. Is the statistical analysis appropriate to answer the research question?
Salvatori, A., 2010 [68]	Yes	No	Yes	Yes	No	Can’t tell	No
Brown, D. et al., 2014 [74]	Yes	No	Yes	Yes	No	Can’t tell	No
Si, W., 2021 [76]	Yes	Yes	Yes	Yes	Can’t tell	No	Yes
	**Screening Questions**		**5. Mixed Methods Studies**				
	S1. Are there clear research questions?	S2. Do the collected data allow to address the research questions?	5.1. Is there an adequate rationale for using a mixed methods design to address the research question?	5.2. Are the different components of the study effectively integrated to answer the research question?	5.3. Are the outputs of the integration of qualitative and quantitative components adequately interpreted?	5.4. Are divergences and inconsistencies between quantitative and qualitative results adequately addressed?	5.5. Do the different components of the study adhere to the quality criteria of each tradition of the methods involved?
Rothboeck, S. et al., 2018 [72]	Yes	Yes	No	Yes	Yes	No	No

This table uses the same categories and format of the MMAT tool [63]. The 11 studies are grouped into five categories according to study design: Qualitative studies ×5, Randomized controlled trials ×1, Non-randomized studies ×1, Quantitative descriptive studies ×3, and Mixed methods studies ×1. The quality assessment questions are slightly different for each study design but have the same answer options: Yes, No, and Can’t tell.

## Data Availability

Data supporting reported results can be obtained from the corresponding author.
